# Primary nodular pulmonary amyloidosis: A case report

**DOI:** 10.1097/MD.0000000000046292

**Published:** 2025-12-19

**Authors:** Chuchu Xu, Xiaoqiong Wang, Xiaona Yin, Xi Wang, Xiaoyue Liu, Dongmei Su, Fangbin Du, Yongsheng Wang

**Affiliations:** a Department of Respiratory and Critical Care Medicine, The Second People’s Hospital of Hefei, Hefei Hospital Affiliated to Anhui Medical University, Hefei, Anhui, China; b Department of Respiratory and Critical Care Medicine, Hefei Second People’s Hospital, Bengbu Medical University, Hefei, Anhui, China.

**Keywords:** asymptomatic, Congo red staining, CT, primary nodular pulmonary amyloidosis

## Abstract

**Rationale:**

This section discusses the etiology, pathogenesis, clinical manifestations, diagnosis, treatment, and prognosis of primary nodular pulmonary amyloidosis.

**Patient concerns:**

We conducted a retrospective analysis of a 60-year-old female patient at Shanghai Chest Hospital who was diagnosed with primary polymorphic pulmonary amyloidosis following the detection of pulmonary nodules during a chest computed tomography (CT) scan as part of a routine physical examination. The analysis encompassed clinical symptoms, imaging and laboratory findings, bronchoscopy results, and medication history.

**Diagnosis:**

Primary nodular pulmonary amyloidosis

**Interventions:**

The patient presented no clinical symptoms. A chest CT scan performed during physical examination revealed pulmonary nodules. These nodules were located extra-luminal. Pathological tissue was obtained via transbronchial needle aspiration under bronchoscopic guidance, utilizing a combination of cone beam computed tomography and rpEBUS. Pathological findings demonstrated polymorphic plasma cell infiltration and multinucleated giant cell proliferation within the pulmonary interstitium, accompanied by deposition of eosinophilic amorphous material. Congo red staining yielded a positive result.

**Outcomes:**

Primary nodular pulmonary amyloidosis lacks specific clinical and imaging manifestations and requires pathological examination and Congo red staining to confirm the diagnosis. There is no recognized treatment option, but early diagnosis and treatment have a certain role in prognosis.

**Lessons:**

Primary nodular pulmonary amyloidosis may present asymptomatically and requires confirmation through a rigorous diagnostic process. Although treatment options are limited, early detection remains crucial for optimizing patient prognosis.

## 1. Introduction

Amyloidosis is a heterogeneous group of diseases characterized by the extracellular accumulation of abnormally folded proteins, leading to organ damage and dysfunction.^[1]^ Lung amyloidosis can manifest in 3 forms: nodular, diffuse alveolar-septal, or tracheobronchial.^[2]^ Clinical symptoms are nonspecific and vary based on the site of involvement, ranging from dyspnea and cough to signs of bronchial obstruction. We report a case involving a 60-year-old patient diagnosed with primary nodular pulmonary amyloidosis. Primary nodular pulmonary amyloidosis is a rare benign pulmonary condition with unclear etiology and pathogenesis. It lacks specific clinical manifestations or imaging findings, making pathological examination and Congo red staining the gold standard for diagnosis. In this article, we present a case of primary nodular pulmonary amyloidosis diagnosed at Shanghai Chest Hospital and describe the clinical features of the disease to enhance physicians’ understanding of this condition.

## 2. Case presentation

**Figure 1.**
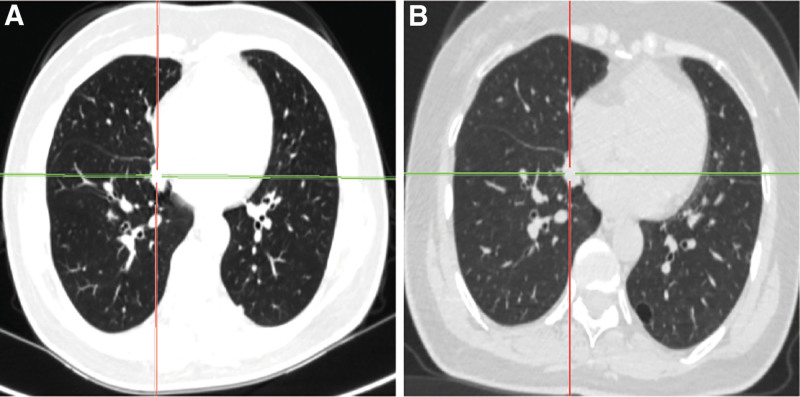
(A) January 2024 chest CT lung window; (B) April 2024 chest CT lung window. CT = computed tomography.

**Figure 2.**
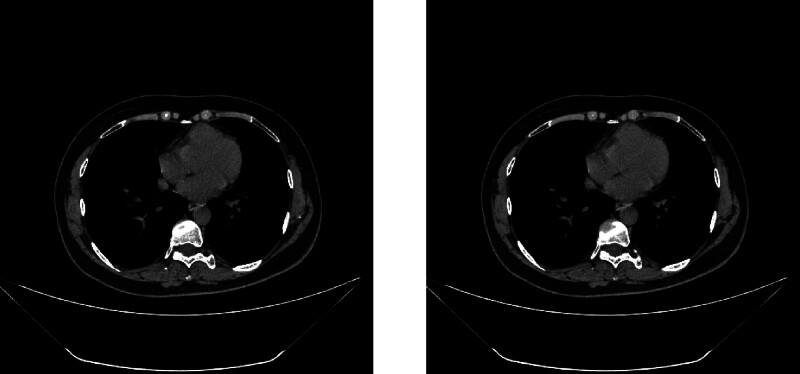
No enlarged lymph nodes or calcifications were observed in the mediastinal window.

**Figure 3.**
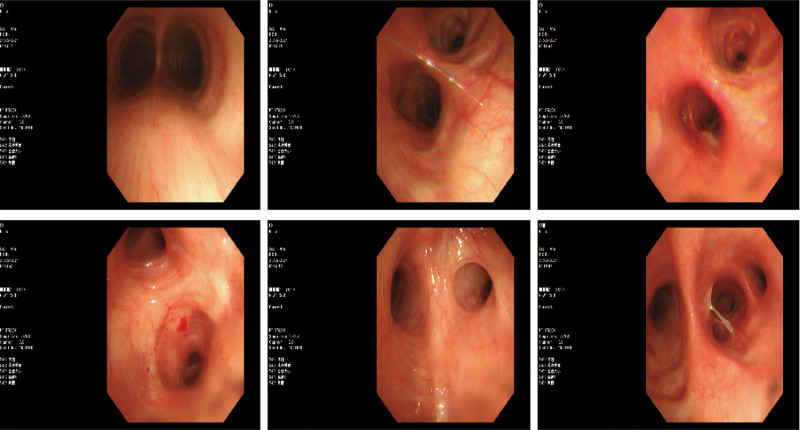
Painless endoscopy report shows that the trachea, right and left common branches, and the lumen of each lobe segment are patent, with smooth mucosa.

**Figure 4.**
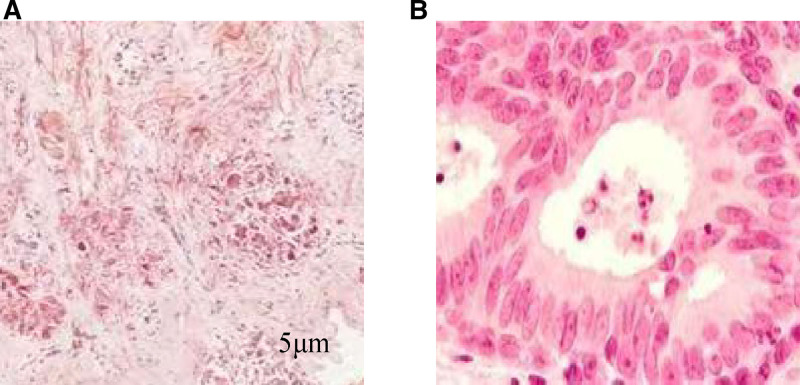
(A) Under the microscope, no structured eosinophilic material is observed (HE ×100); (B) Congolese red staining positive.

A 60-year-old female patient of Han ethnicity, employed as a farmer, has no documented history of occupational or environmental exposures, nor any prior smoking or alcohol consumption. Additionally, there is no family history of hereditary diseases. She was admitted to the hospital due to “two lung nodules identified over the past 3 years.” A CT scan performed on the patient in 2021 revealed nodules in both lungs, which have been regularly monitored. A chest CT scan conducted in February 2023 indicated the presence of new nodules in the upper lobe of the left lung compared to previous scans. In the chest CT scan conducted on January 2024, multiple nodular high-density shadows were observed in both lungs, with the largest measuring approximately 13 mm × 10 mm, located in the basal segment of the right lower lobe (Fig. 1A). The patient underwent a 2-week course of anti-infective therapy, and a follow-up chest computed tomography (CT) scan with contrast performed 3 months later revealed no significant changes in the lesion (Fig. 1B). Advanced CT demonstrated slight enhancement of the nodule, the absence of high attenuation in the mediastinal view, and no enlargement of the lymph nodes (Fig. 2). The patient’s autoimmune system-related tests, including assessments of the rheumatic system, antinuclear antibody, anti-neutrophil antibody, cardiac echocardiogram, and echocardiograms of the liver, gallbladder, pancreas, spleen, and kidneys, showed no abnormalities. To further characterize the nature of the lung lesions, we performed a painless bronchoscopy. The bronchoscopy report indicated that the lumens of the trachea, right and left main bronchi, and all lobar segments were patent, with smooth mucosa (Fig. 3). The pulmonary nodule in this patient is located outside the bronchial lumen, making bronchoscopic access to the nodule challenging. We employed a transpulmonary bronchoscopic approach (bronchoscopic transparenchymal nodule access) to reach the nodule. This involved creating a hole in the bronchial wall, establishing a tunnel, and accessing the nodule via a working channel within the lung parenchyma. Following real-time localization using a combined radial probe EBUS and cone beam computed tomography (CBCT), biopsies were obtained from the medial and lateral basal segments of the right lower lobe. Pathological examination revealed: extensive plasma cell infiltration and multinucleated giant cell proliferation within the pulmonary interstitium, accompanied by eosinophilic amorphous deposits. Immunohistochemical results demonstrated positivity for: creatine kinase (epithelial cells+), TTF-1 (epithelial cells+), and Napsin A (epithelial cells+), while P40 (−), and CD56 (−) were negative. Supplementary immunohistochemistry revealed: CD34 (vascular +), ERG (−), IgG protein (+), IgG4 protein (−); (Fig. 4A). These negative immunohistochemical results exclude epithelial tumors and malignant pulmonary neoplasms. Special stains demonstrated: Congo red staining (weakly positive) and methyl violet staining (positive; Fig. 4B). Considering the combination of specialty stains, pulmonary amyloidosis was suggested. The patient had previously been healthy and reported no characteristic discomfort. No tumor cells were identified in 2 pathological examinations, effectively ruling out secondary pulmonary amyloidosis. The diagnosis of primary nodular pulmonary amyloidosis was confirmed, with no associated connective tissue diseases present. Unfortunately, subsequent patients, who resided outside the local area, were followed up at local hospitals, and no further follow-up examination materials were available.

## 3. Discussion

It is classified into 3 categories based on the distribution of deposited amyloid: bronchiolar pulmonary amyloidosis, which is primarily confined to the trachea and peribronchioles; nodular pulmonary amyloidosis, characterized by the formation of tumoral nodules; and interstitial-vascular diffuse alveolar septal amyloidosis, which refers to pulmonary deposition occurring within the interstitial walls and vascular media.^[2]^ Many cases of nodular pulmonary amyloidosis, particularly asymptomatic cases, are incidentally detected through imaging.^[3]^ Characteristic CT findings of pulmonary amyloidosis nodules include multiple irregular nodules, approximately 3 cm in size, often associated with calcifications, cavities, and cysts. These nodules may exhibit a tendency to increase in size.^[4]^ CT nodules are predominantly distributed in the lower lungs, subpleura, or periphery of the lungs, and they often present as single or multiple round or round-like nodules. The size of the nodules ranges from 0.4 to 15 cm, with edges that are typically smooth. Some of the foci may exhibit enhancement. They can also present as multiple high-density, corn-grain-like foci within the lungs, which may indicate the deposition of amyloid in the lung interstitial space. Imaging studies are essential for diagnosing pulmonary amyloidosis, providing critical evidence for subsequent treatment and further examinations. The imaging characteristics of pulmonary nodular amyloidosis can be summarized as follows: After excluding secondary pulmonary amyloidosis, approximately 60% of patients present with solitary tumor-like deposits. Nodules are primarily located in the lower lobes of the lungs, distributed sporadically, and are commonly found in subpleural or peripheral regions. Nodules or masses exhibit diverse morphologies, potentially presenting smooth margins, lobulated patterns, or spiculated features. Approximately 50% of cases reported in the literature demonstrate significant calcification or ossification. In some instances, pulmonary cysts may accompany pulmonary nodules, and amyloid protein deposits may lead to gas retention, fragile alveolar walls, or alveolar wall damage due to ischemia. Imaging monitoring indicates that the progression of pulmonary nodules is typically slow. Chest CT plays a vital clinical role in assessing disease severity, classification, treatment efficacy, and guiding therapeutic interventions.

The physical examination of the patient revealed the presence of asymptomatic lung nodules. However, subsequent evaluations indicated an increase in the size of these nodules, raising concerns about potential infectious or malignant processes. Following standard anti-infective treatment and a review of chest CT scans that showed no significant changes, infectious etiologies were ruled out temporarily, although malignancy could not be entirely excluded. Two biopsies were performed, targeting the inner and outer basal segments of the right lower lobe, using precise guidance from CBCT and ultrasonography, which ultimately identified a neoplastic basis. Pathological analysis of both biopsies indicated a proliferation of multinucleated giant cells, suggesting a process consistent with pulmonary amyloidosis, potentially accompanied by chronic inflammatory cell infiltration, particularly involving macrophages and lymphocytes. To further characterize the lesions, specific staining techniques, including Congo red and methyl violet staining, were employed, ultimately confirming the diagnosis of pulmonary amyloid deposition disease. Based on the common clinical manifestations of pulmonary amyloidosis, the following differential diagnoses should be considered – primary lung cancer/metastatic cancer: imaging studies can rule out metastatic cancer characterized by multiple nodules and calcification; however, diffuse tracheobronchial wall thickening with calcification is atypical. Central-type lung cancer generally presents as unilateral bronchial obstruction rather than bilateral wall thickening. Bronchoscopy did not reveal typical cauliflower-like, papillary, or ulcerative masses; the mucosal surface appeared relatively smooth but exhibited hard calcifications. Pathological examination did not identify malignant epithelial cells, sarcoma cells, or evidence of metastatic cancer. Immunohistochemistry results showed TTF-1 positivity (epithelial type+), P40 negativity, and CD56 negativity, thus ruling out pulmonary malignant tumors. Lymphoma: Imaging studies may demonstrate thickening or nodules of the bronchial wall, but diffuse calcification is rare. Mucosal thickening observed during bronchoscopy is typically soft, and calcification is uncommon. Pathological examination did not reveal diffuse infiltration of lymphocytes or characteristic lymphoepithelial lesions; Congo red staining was positive, confirming the presence of amyloid material rather than lymphocyte proliferation. Mycobacterial tuberculosis infection and fungal infection: Imaging studies may reveal calcified nodules and lymph node calcification. Tuberculosis may present with diffuse thickening of the tracheobronchial walls with calcification, often accompanied by ulcers, granulomas, and scarring, with cavities being more prevalent. Calcified nodules are commonly observed in fungal infections; however, diffuse airway wall calcification is considered atypical. Bronchoscopy did not reveal ulcers, active granulomas, or caseous necrosis – findings typically associated with tuberculosis – nor did it detect fungal hyphae or yeast cells. Pathological examination also failed to show caseous necrosis, acid-fast bacilli, or fungi, and did not reveal granulomatous structures.

In the case of sarcoidosis, typical radiological findings include bilateral hilar lymphadenopathy (with calcification being rare) and small nodules surrounding the lymphatic vessels. The extent of lymph node calcification in sarcoidosis is less pronounced than in amyloidosis or pneumoconiosis. Although bronchoscopy may demonstrate narrowing due to lymph node enlargement compressing the bronchi or granulomatous infiltration, direct causes of widespread airway wall calcification are infrequent. Pathological examination did not reveal noncaseating epithelioid cell granulomas. Regarding pulmonary hamartoma/chondroma, imaging typically excludes solitary, well-defined nodules containing fat or calcification. These do not present with diffuse tracheobronchial wall thickening and calcification or multiple diffuse pulmonary nodules. In terms of bronchoscopy and pathology exclusion, bronchial-type hamartomas may obstruct the bronchi; however, microscopic and pathological examinations reveal mixed tissue components, such as cartilage, fat, and epithelium, rather than homogeneous amyloid material.

The clinical manifestations of pulmonary amyloidosis are closely related to the severity and location of the lesion. Diffuse alveolar-septal or tracheobronchial amyloidosis, which can directly threaten the patient’s life, often presents with a variety of symptoms. These symptoms include severe cough, hemoptysis, dyspnea, shortness of breath, and even the gradual development of respiratory failure.^[5,6]^ Pulmonary nodular amyloidosis in the early stage typically presents with no obvious respiratory symptoms and is often discovered during physical examinations or autopsies. In this particular case, the patient did not exhibit any clinical symptoms and the nodules were detected during a physical examination. Bronchoscopy was performed to determine the nature of the lesion. While pulmonary nodular amyloidosis can sometimes lead to hemoptysis or even pneumothorax, these symptoms are rare, mild, and nonspecific. Overall, this type of pulmonary amyloidosis has the most favorable prognosis among all described diseases.^[7]^

Histopathological analysis of collected samples is essential for establishing a diagnosis of amyloidosis. A key and characteristic finding from this examination is the presence of apple-green birefringence under polarized light following Congo red staining. Subsequent analysis is primarily conducted through immunohistochemical methods to accurately identify the specific type of amyloidosis.^[8,9]^ Primary nodular pulmonary amyloidosis currently lacks an effective treatment. Management options, including surgical resection, percutaneous lung puncture ablation, endoscopic freezing, laser treatment, and symptomatic management, are selected based on the patient’s symptoms, lesion size, and location. For patients with tracheobronchial and diffuse alveolar septal types of pulmonary amyloidosis, symptoms may include cough, hemoptysis, and shortness of breath. Additional treatment options may consist of transbronchoscopic removal, pharmacotherapy, and symptomatic treatment. In cases of nodular pulmonary amyloidosis, asymptomatic patients may be monitored regularly.^[10]^ If the nodule increases in size or complications arise during follow-up, surgical resection may be considered.^[11]^ Currently, there is no standardized treatment protocol for this condition, particularly for asymptomatic patients or those presenting with mild symptoms and localized lesions. A consensus on the optimal management strategy is lacking. Treatment options are limited, and the supporting evidence is insufficient. For bronchial lesions, interventions such as laser therapy, cryotherapy, and stent placement can alleviate obstructive symptoms; however, these methods are associated with a risk of recurrence and do not address the underlying issue. Additionally, sensitive or specific biomarkers to assess treatment efficacy are absent, and evaluation primarily relies on imaging studies (CT), pulmonary function tests, and symptomatic changes, which may occur slowly or be negligible.

The progressive enlargement of pulmonary nodules (>2 mm/year) typically raises concerns about malignancy. Key diagnostic pitfalls include: misinterpreting granulomatous inflammation as sarcoidosis despite atypical distribution; overlooking indolent infections in immunocompetent patients; and sampling errors in small nodules. Serial imaging and multidisciplinary review are critical to avoid misdiagnosis.

Primary nodular pulmonary amyloidosis cases are not uncommon; however, our report is distinctive due to the presence of multiple pulmonary nodules visible on CT scans without calcification, which differs from previously documented cases. Our patient exhibited no clinical symptoms of systemic lupus erythematosus or other autoimmune diseases. The nodules were detected during a routine physical examination, and the patient remained asymptomatic. Initially, no intervention was performed, but follow-up revealed that some nodules had enlarged. Standard antimicrobial therapy resulted in no significant changes, and 2 bronchoscopic biopsies failed to detect tumor cells.

A unique aspect of our case is the method employed to obtain pathological tissue samples. Typically, researchers utilize CT-guided percutaneous lung biopsy or lung wedge resection. However, our patient’s nodules were not subpleural, rendering percutaneous biopsy impractical. Based on chest CT findings, we determined that the lesion was outside the bronchial lumen and did not pass through it. With the patient’s consent, we successfully obtained pathological tissue using bronchoscopic transparenchymal nodule access combined with CBCT and rpEBUS.

## 4. Conclusion

Primary pulmonary amyloidosis is a rare condition characterized by nonspecific clinical symptoms, imaging findings, and bronchoscopic results, which often lead to misdiagnosis as lung cancer or granulomatous diseases. Diagnosis primarily depends on pathological biopsy, specific staining techniques, and immunohistochemical analysis. The main diagnostic challenge is to identify amyloidosis and accurately determine whether it is a primary localized condition or a local manifestation of a systemic disease. In terms of management, the lack of evidence-based guidelines necessitates individualized and multidisciplinary decision-making. This case underscores the importance of expanding diagnostic considerations when encountering atypical pulmonary lesions, actively obtaining high-quality pathological diagnoses, and conducting comprehensive evaluations to ensure that patients receive precise treatments and effective prognostic assessments.

## Author contributions

**Conceptualization:** Xiaoqiong Wang.

**Data curation:** Xiaona Yin, Xi Wang, Xiaoyue Liu, Fangbin Du.

**Formal analysis:** Xi Wang, Dongmei Su.

**Writing – original draft:** Chuchu Xu.

**Writing – review & editing:** Xiaoqiong Wang, Yongsheng Wang.
